# Exploring spatiotemporal trends and impacts of health resources and services on under-5 mortality in West African countries, 2010–2019: a spatial data analysis

**DOI:** 10.3389/fpubh.2023.1193319

**Published:** 2023-09-13

**Authors:** Meng Zeng, Lu Niu

**Affiliations:** Department of Social Medicine and Health Management, Xiangya School of Public Health, Central South University, Changsha, China

**Keywords:** under-5 mortality, health resources and services, spatiotemporal trend, spatial spillover effect, spatial regression, West Africa

## Abstract

**Background:**

West African countries experienced the highest under-5 mortality rate (U5MR), the lowest life expectancy, and the poorest economic development. This study aimed to explore the spatiotemporal trend of U5MR and spatial spillover effects of health resources and services to provide a basis for establishing health policies and international cooperative mechanisms in West Africa.

**Methods:**

We retrieved data from the World Health Organization’s Global Health Observatory, the United Nations Human Development Report, and the Global Burden of Disease Study 2019. Joinpoint regression analysis and Moran’s *I* method were used to examine the temporal trend and spatial dependence of U5MR, respectively. Spatial regression analysis was used to examine the spatial spillover effects.

**Results:**

The long-term downward trends in U5MR were divided into multiple segments by two or three change points in 2013, 2014, or 2015, and the annual percentage change after 2015 was higher than before 2015. Global Moran’s *I* was positive, significantly indicating positive spatial autocorrelation, which increased from 0.2850 (*p* = 0.0210) to 0.3597 (*p* = 0.0080). Based on spatial regression analysis, human development index (HDI), physicians density, nurses and midwives density, health center density, percentage of infants lacking immunization for diphtheria and measles, and coverage rate of at least one antenatal visit had negative spatial spillover effects on U5MR. HDI had the strongest negative correlation (*β* = −0.0187 to −0.1054, *p* < 0.0001). Current health expenditure (CHE) *per capita* had positive spatial spillover effects on U5MR.

**Conclusion:**

This study revealed the spatiotemporal trend of U5MR in West African countries and spatial spillover effects of health resources and services. Promoting economic development, increasing health human resources, health expenditure, vaccination rate, antenatal care coverage, and the proportion of health professionals attending births not only reduced the local U5MR but also exerted spatial spillover effects on adjacent countries. The West African Health Organization may consider regional spillover mechanisms to develop regional health policy and intervention cooperation mechanisms, which will contribute to achieving the sustainable development goal on U5MR, Africa Agenda 2063, and universal health coverage.

## 1. Introduction

The under-5 mortality rate (U5MR) refers to the deaths per 1,000 live births under 5 years of age. It is an important statistic for global children’s health and well-being, i.e., access to nutrition and food, housing, and infrastructure, such as water and sanitation, education, property security, access to preventive and curative health services, and future human capital (1). United Nations Children’s Fund (UNICEF) and World Bank have considered reducing child mortality as part of future strategic planning. In addition, reducing U5MR was the fourth Millennium Development Goal (2). The third Sustainable Development Goal (SDG 3) is to reduce the U5MR deaths at least to 25 deaths per 1,000 live 50 births in all countries by 2030 (3).

Despite global efforts to achieve SDG 3 by improving population health over time, child health remains poor in several countries, and geographic inequalities persist between countries with the lowest and highest child mortality rates. The Global Burden of Disease Study 2019 indicated that although global U5MR decreased from 71.2 [95% confidence interval (CI): 68.3–74.0] per 1,000 live births in 2000 to 37.1 (95% CI: 33.2–41.7) per 1,000 live births in 2019, it remained the highest in sub-Saharan Africa, with 74.1 (95% CI: 65.3–85.2) per 1,000 live births in 2019 (1). Moreover, despite that progress, West Africa experienced the highest U5MR with 95.3 per 1,000 live births in 2019 (95% CI: 84.7–109) (1). In addition, the healthy life expectancy was relatively the lowest, and economic development was the poorest in most West African countries (4). Evidently, West African countries are experiencing poorer child health and facing more serious challenges in achieving the SDG on U5MR than other countries. Reducing U5MR and preventing deaths in children has become a global consensus (5), even more urgent for West African countries that are making low progress.

Previous studies have indicated the existence of spatiotemporal variations and identified high U5MR clusters, patterns of progress and geographical inequalities (6). Spatial spillovers are widely recognized in explaining the spatial dependence of subjects to some extent, for example, life expectancy (7) or population aging (8). These studies estimated that three-quarters of the variations can be attributed to temporal or spatial differences between countries in Sub-Saharan Africa (9). For example, an ecological study of China investigated the relationship between the U5MR, geographic, and socioeconomic factors and explored the associated spatial spillovers of the relationship, indicated that the development of economic and medical standards can overcome geographical limits (10). The socioeconomic development in sub-Saharan Africa impacted differently on U5MR, such as the mother’s education level, child sex, family wealth level, and residence (11). In addition, provision of health resources and services guarantees the promotion of children’s health. A previous study found that inequality of health resource distribution was significantly associated with higher U5MR, while the quantity of health resources had a statistically insignificant inverse association with U5MR (12). Health resources refer to various factors of production used by the society in providing medical and health services, including human and material resources (12). Health service is a fundamental input to population health status, along with other factors, including social determinants of health (13).Health resources and services were closely related to the U5MR, the core indicators of health system monitoring by the World Health Organization and the United Nations SDG 3c, i.e., the density of physicians, nurses and midwives, and the density of health facilities (3, 14). An ecological study of 46 African countries indicated that promoting socioeconomic development and increasing coverage of maternal health service and vaccination rates exhibited significant negative effects on U5MR (15). Moreover, other prior studies found that health expenditure impacted child health, with a negative correlation between health expenditure, infant mortality rate, and U5MR in West Africa (16). Two non-spatial ecological studies illustrated that increasing the proportion of births attended by skilled professionals, the coverage of diphtheria vaccination, measles vaccination, etc., may improve U5MR (17, 18). Nevertheless, it is important to understand the relationship between geographical elements and the U5MR in space, since different regions have different health resources and services characteristics. However, due to the lack of the refined long-term U5MR data and the gridded data for geographical and health resource and services factors, studies on the spatial relationship between them have seldom been conducted in West Africa.

Spatial spillovers are widely recognized in explaining the spatial dependence of U5MR to some extent and provide an empirical basis for establishing international health cooperation, regional health cooperation, and health resource allocation mechanism. Analyzing the spatiotemporal characteristics of U5MR and the spatial spillover effect of health resources and services can comprehensively explain the long-term trend of health inequality in different countries in this region. It is of great significance to guide regional health planning and formulate regional health policies in West Africa, conducive to reducing child health inequality between and within countries (19). Hence, the present study aimed to describe the spatiotemporal characteristics of U5MR in 15 West African countries from 2010 to 2019 by combining the ecological design of geographic information systems and explore the spatial spillover effects of health resources and services on U5MR from the perspective of spatiotemporal correlation.

## 2. Materials and methods

### 2.1. Indicators and data sources

The U5MR, which is the deaths per 1,000 live births under 5 years of age, is the core measure of child health. Data on U5MRs were retrieved from the Global Burden of Disease Study 2019 (GBD2019, https://www.healthdata.org/gbd/2019), which was led by the Institute for Health Metrics and Evaluation at the University of Washington that provides rigorous and comparable measurements of the world’s most important health problems and evaluates the strategies used to address them. In this study, West African countries referred to the 15 member countries of the Economic Community of West African States (ECOWAS), including Benin, Burkina Faso, Cote d’Ivoire, Cape Verde, Gambia, Ghana, Guinea, Guinea-Bissau, Liberia, Mali, Niger, Nigeria, Sierra Leone, Senegal, and Togo.

#### 2.1.1. Socioeconomic development indicators

The human development index (HDI) was the selected indicator in this study to reflect socioeconomic development at the country level. The HDI is the comprehensive indicator used to assess the health and quality of life of a population, including education, health, and income, which was first published by the United Nations Development Program^1^ in 1990. It was calculated in three parts, including adult literacy to represent education, life expectancy at birth to represent health, and gross domestic product *per capita* to represent income (20).

#### 2.1.2. Health resources and services indicators

It is well known that economic development, health policies, and governance strategies have significant effects on population health. This is especially evident when examining health systems, such as the inequitable distribution of health resources and services, which hindered social development and affected population health (21). Based on the literature and the WHO prison health framework: a framework for assessment of prison health system performance (13, 21), health resources and services indicators include physicians density, nurses and midwives density, pharmaceutical personnel density, health center density, *per capita* health expenditure, percentage of infants lacking immunization for diphtheria and measles, birth registration rate of children under 5 years of age, coverage rate of at least one antenatal visit, contraceptive rate of women of reproductive age 15–49 years, and proportion of births attended by skilled health personnel. Data are available on health resources and services indicators from the Global Burden of Disease Study 2019 (GBD2019, https://www.healthdata.org/gbd/2019), Global Health Observatory (WGHO, https://www.who.int/data/gho), Human Development Report (HDR, see footnote 1; Table 1).

**Table 1:** Descriptions of under-5 mortality rate and health resources and services indicators.

	No.	Variables	Definition	Unit	Data source
Health outcome	Y	Under-5 mortality rate	The number of deaths of a child born in a specific year or period dying before reaching the age of 5 years divided by the number of population at risk during a certain period of time	Per 100,000 population	GBD2019^a^
Socioeconomic	X1	Human development index	A composite index measuring average achievement in three basic dimensions of human development long and healthy life, knowledge and a decent standard of living	0–1	HDR^b^
Health resources	X2	Physicians per 10,000 population	The number of physicians available in a country relative to the total population	Per 10,000 population	GBD2019
	X3	Nurses and midwives per 10,000 population	The number of nurses and midwives available in a country relative to the total population	Per 10,000 population	GBD2019
	X4	Pharmaceutical personnel per 10,000 population	The number of pharmaceutical personnel available in a country relative to the total population	Per 10,000 population	GBD2019
	X5	Health centers per 10,000 population	The number of health centers available in a country relative to the total population	Per 10,000 population	WGHO^c^
	X6	Current health expenditure (CHE) *per capita* in US$	Spending on healthcare goods and services, expressed as *per capita* in US$	US$	WGHO
Health services	X7	Infants lacking immunization, DTP (% of one-year-olds)	Percentage of surviving infants who have not received their first dose of diphtheria, tetanus and pertussis vaccine	%	HDR
	X8	Infants lacking immunization, measles (% of one-year-olds)	Percentage of surviving infants who have not received the first dose of measles vaccine	%	HDR
	X9	Birth registration (% under age 5)	Proportion of children under 5 years of age whose births have been registered with a civil authority	%	HDR
	X10	Antenatal care coverage, at least one visit (%)	Percentage of women ages 15**–**49 attended at least once during pregnancy by skilled health personnel (doctor, nurse or midwife)	%	HDR
	X11	Contraceptive prevalence, any method (% of married or in-union women of reproductive age, 15–49 years)	Percentage of married or in-union women of reproductive age (15**–**49 years) currently using any contraceptive method	%	HDR
	X12	Proportion of births attended by skilled health personnel (%)	Percentage of childbirths attended by skilled health personnel (generally doctors, nurses or midwives) who are maternal and newborn health professionals educated, trained and regulated to national and international standards	%	HDR

a GBD2019, Global Burden of Disease 2019.

b HDR, Human development report.

c WGHO, World Health Organization’s Global Health Observatory.

### 2.2. Statistical analysis

#### 2.2.1. Joinpoint regression model

The joinpoint regression model is widely used to analyze the trend of morbidity or mortality of tumors and chronic diseases over time (22). This model can divide the longitudinal variations into different segments by piecewise regression and identify the segment trends with statistical significance. Temporal trends in West African countries where U5MR were measured by the annual percentage change (APC) and average annual percentage change (AAPC), with 95% CIs, using joinpoint regression models (22). To determine the magnitude of the temporal trends for mortality rates, we used a permutation procedure to divide the long-term change trend of mortality rate into several segments by determining the numbers of change points, the AAPCs, and the corresponding 95% CIs were evaluated by joinpoint regression analysis. When describing temporal trends, the terms increase and decrease indicate that the slope (APC and AAPC) was significant at the 0.05 level (23). This analysis was performed using Joinpoint software version 4.9.1.0 from the Surveillance Research Program of the US National Cancer Institute.^2^

#### 2.2.2. Global and local spatial autocorrelation

Spatial autocorrelation analysis was performed using ArcGIS version 10.7 (Environmental Systems Research Institute Inc., Redlands, CA), and the spatial clustering of U5MR was performed by calculating Moran’s *I*. The value range of I is [−1, 1], *I* > 0, and is significant at the 0.05 level, indicating the presence of positive spatial autocorrelation. The spatial autocorrelation was calculated using the local indicators of spatial association (LISA) analysis. This index explains the spatial relationship pattern of a spatial parameter in the neighborhood. The global and local Moran’s *I* can be calculated as follows (24):


(1) $$I=\frac{n\sum_{i=1}^{n}\sum_{j=1}^{n}w_{ij}\left(x_{i}-\bar{x}\right)\left(x_{j}-\bar{x}\right)}{\sum_{i=1}^{n}\sum_{j=1}^{n}w_{ij}\sum_{i=1}^{n}{\left(x_{i}-\bar{x}\right)}^{2}}$$

(2) $$I_{i}=Z_{i}\underset{j}{\sum }W_{ij}Z_{j}$$

where *n* represents the number of countries, $x_{i}$ and $x_{j}$ refer to the U5MRs of countries *i* and *j*, respectively, and $w_{ij}$ represents the spatial weight matrix, the adjacency relationship between countries *i* and *j*, with 1 for adjacency and 0 for non-adjacency. $Z_{i}$ and $Z_{j}$ represent the deviations of the U5MRs from the mean for countries *i* and *j*, respectively, and $Z_{i}=\frac{X_{i}-\bar{X}}{\delta }$.

#### 2.2.3. Spatial regression analysis

The ordinary linear regression model (OLS) is the basis of spatial regression analysis, lacking consideration of the spatial weight matrix and interpretation of the spatial autocorrelation or spatial lag effect. In comparison, spatial regression models take spatial autocorrelation and dependence into account. The spatial regression model analysis, including the spatial error model (SEM) and spatial lag model (SLM), was performed in our study to explore the influence factors on U5MR in West African countries (25). The SEM assumes that there is spatial autocorrelation from errors between explanatory variables in adjacent study areas. The SLM explains autocorrelation by adding a lagged dependent variable of one region that is related to its neighbors. The SEM can be expressed as follows:


(3) Y=Xβ+ε

(4) $$\varepsilon =\lambda W_{\varepsilon }+\mu$$

where *Y* is the dependent variable, representing the U5MR; *X* is an independent variable (including constant), representing socioeconomic and health resource indicators; *β* refers to the estimated parameter; *W* represents the normalized spatial weight matrix; *λ* represents the spatial autocorrelation parameter; $W_{\varepsilon }$ represents the spatial error; and *ε* represents the random error that follows a normal distribution.

The SLM can be expressed as follows:


(5) $$Y=X\beta +\rho W_{y}+\varepsilon_{0}$$

where *Y* is the dependent variable, representing the U5MR; *X* refers to an independent variable (including constant), representing socioeconomic and health resource indicators; *β* represents the spatial regression coefficient of the independent variable; *ρ* represents the spatial autocorrelation parameter; and $\varepsilon_{0}$ represents the random error that follows a normal distribution.

After confirming the spatial regression model, the Lagrange multiplier (LM) test was used to select the SLM and SEM. The LM test includes the LM-error test proposed by Bridge in 1980 (26), the LM-lag test proposed by Anselin in 1988 (27), and the robust LM-lag test and the robust LM-error test proposed by Bela and Ido in 1922. Akaike information criterion (AIC), Schwarz criterion (SC), log-likelihood, and variance were used to compare the goodness-of-fit degree of the two spatial regression models. The smaller the AIC and SC, the larger the log-likelihood and variance, and the better the model fit. This study determined that a two-sided test with *α* < 0.05 was statistically significant. Using inverse distance spatial weights, the sensitivity analysis changed the “Queen contiguity” matrix to a “Rook” matrix of adjacent points using GeoDa 1.18.

## 3. Results

### 3.1. Spatiotemporal trends in West African countries, 2010–2019

**Figure 1:**
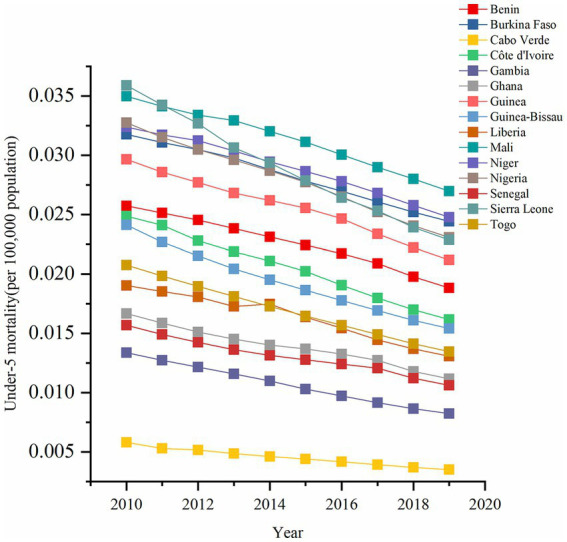
Temporal trend of under-5 mortality rate (per 100,000 population) from 2010 to 2019 in West African countries.

The temporal trends in U5MR were a long-term consistent decrease during our study period (Figure 1). The results of the joinpoint regression model showed that the numbers of change points were different during 2010–2019, and the trend of each stage decreased (Table 2). The mortality rate significantly and progressively decreased, with all APCs and AAPCs being statistically significant during the whole study period. Two or three change points were observed in most countries in 2013, 2014, or 2015, and long-term temporal trends were divided into segments, except in Cape Verde and Sierra Leone. In addition, the APCs of the post-2015 sub-trend were higher than those of the pre-2015 sub-trend, such as Senegal with a significant APC of −6.1051 (95% CI: −7.5139 to −4.6747) from 2017 to 2019, more evident than 2010–2013 and 2013–2017, indicating the most significant overall decrease with the greatest percentage change of 5.3095% (AAPC 95% CI: −5.4774 to −5.1413) during 2010–2019, and two staged trends included the percentage change of 4.6325% (APC 95% CI: −5.1194 to −4.1431) during 2010–2013 and the percentage change of 5.6462% (APC 95% CI: −5.8699 to −5.422) during 2013–2019 in Gambia. Cape Verde and Ghana were followed directly by Gambia with AAPC of −5.2042 (95% CI: −5.5328 to −4.8744) and AAPC of −4.4127 (95% CI: −4.8265 to −3.9971), respectively. Although Mali experienced the highest U5MR of 0.0270 (95% CI: 0.0220–0.0333) per 100,000 population in 2019, it had the lowest percentage change of 2.7955% (AAPC 95% CI: −2.9377 to −2.6532).

**Table 2:** The annual percentage change (APCs) and average annual percentage rate change (AAPCs) in West African countries.

Country	Period (year)	APC (95% CI)	*t* value	*p*-value	AAPC (95% CI)	*z* value	*p*-value
Benin	2010–2012	−2.3551 (−3.2303 to −1.4719)	−11.3881	0.0076^**^	−3.3973 (−3.5664 to −3.2279)	−38.6677	<0.0001^**^
	2012–2016	−2.9337 (−3.4471 to −2.4176)	−24.1604	0.0017^**^			
	2016–2019	−4.6961 (−5.3479 to −4.0398)	−30.1568	0.0011^**^			
Burkina Faso	2010–2013	−2.1492 (−2.2561 to −2.0422)	−51.1001	<0.0001^**^	−2.8909 (−2.9278 to −2.854)	−151.393	<0.0001^**^
	2013–2019	−3.2596 (−3.3089 to −3.2103)	−167.2006	<0.0001^**^			
Côte d’Ivoire	2010–2015	−4.1602 (−4.5739 to −3.7448)	−25.2515	<0.0001^**^	−4.7440 (−5.0345 to −4.4527)	−31.1936	<0.0001^**^
	2015–2019	−5.4687 (−6.1478 to −4.7848)	−20.0532	<0.0001^**^			
Cabo Verde	2010–2019	−5.2042 (−5.5328 to −4.8744)	−35.4918	<0.0001^**^	−5.2042 (−5.5328 to −4.8744)	−35.4918	<0.0001^**^
Gambia	2010–2013	−4.6325 (−5.1194 to −4.1431)	−23.8196	<0.0001^**^	−5.3095 (−5.4774 to −5.1413)	−60.2612	<0.0001^**^
	2013–2019	−5.6462 (−5.8699 to −5.422)	−62.9337	<0.0001^**^			
Ghana	2010–2012	−4.9395 (−6.4837 to −3.3697)	−13.3075	0.0056^**^	−4.4127 (−4.8265 to −3.9971)	−20.3894	<0.0001^**^
	2012–2017	−3.3014 (−4.0023 to −2.5953)	−19.8548	0.0025^**^			
	2017–2019	−6.6210 (−9.8489 to −3.2775)	−8.3783	0.0139^*^			
Guinea	2010–2016	−2.9740 (−3.2599 to −2.6873)	−26.3025	<0.0001^**^	−3.6357 (−3.9677 to −3.3026)	−21.0328	<0.0001^**^
	2016–2019	−4.9457 (−6.1011 to −3.7761)	−10.6615	0.0001^**^			
Guinea-Bissau	2010–2012	−5.6030 (−6.1403 to −5.0626)	−25.9656	<0.0001^**^	−4.8595 (−4.9615 to −4.7573)	−90.9800	<0.0001^**^
	2012–2019	−4.6460 (−4.7207 to −4.5712)	−155.9723	<0.0001^**^			
Liberia	2010–2014	−2.3885 (−2.989 to −1.7843)	−10.0707	0.0002^**^	−4.1111 (−4.445 to −3.7761)	−23.5896	<0.0001^**^
	2014–2019	−5.4673 (−6.0883 to −4.8422)	−21.9302	<0.0001^**^			
Mali	2010–2014	−2.0525 (−2.3211 to −1.7833)	−19.4189	<0.0001^**^	−2.7955 (−2.9377 to −2.6532)	−37.9732	<0.0001^**^
	2014–2019	−3.3859 (−3.6432 to −3.1279)	−33.2004	<0.0001^**^			
Niger	2010–2012	−1.7620 (−2.0985 to −1.4243)	−22.2892	0.0020^**^	−2.9100 (−2.9708 to −2.849)	−92.2765	<0.0001^**^
	2012–2016	−2.8402 (−3.0282 to −2.6519)	−64.0209	0.0002^**^			
	2016–2019	−3.7600 (−3.9785 to −3.5411)	−72.5605	0.0002^**^			
Nigeria	2010–2015	−3.2241 (−3.45 to −2.9977)	−36.0483	<0.0001^**^	−3.8110 (−4.0157 to −3.6059)	−35.7513	<0.0001^**^
	2015–2019	−4.5395 (−5.0694 to −4.0067)	−21.4565	<0.0001^**^			
Senegal	2010–2013	−4.6392 (−4.9472 to −4.3302)	−63.1726	0.0003^**^	−4.2512 (−4.4346 to −4.0675)	−44.4085	<0.0001^**^
	2013–2017	−3.0157 (−3.5152 to −2.5137)	−25.5179	0.0015^**^			
	2017–2019	−6.1051 (−7.5139 to −4.6747)	−17.9279	0.0031^**^			
Sierra Leone	2010–2019	−4.9404 (−5.0563 to −4.8243)	−95.725	<0.0001^**^	−4.9404 (−5.0563 to −4.8243)	−95.7250	<0.0001^**^
Togo	2010–2014	−4.4617 (−4.5742 to −4.349)	−99.5346	<0.0001^**^	−4.6990 (−4.7618 to −4.6361)	−143.0299	<0.0001^**^
	2014–2019	−4.8884 (−5.0062 to −4.7705)	−103.9882	<0.0001^**^			

APC, annual percentage change; AAPC, average annual percentage rate change. ^*^p < 0.05, ^**^p < 0.01.

**Figure 2:**
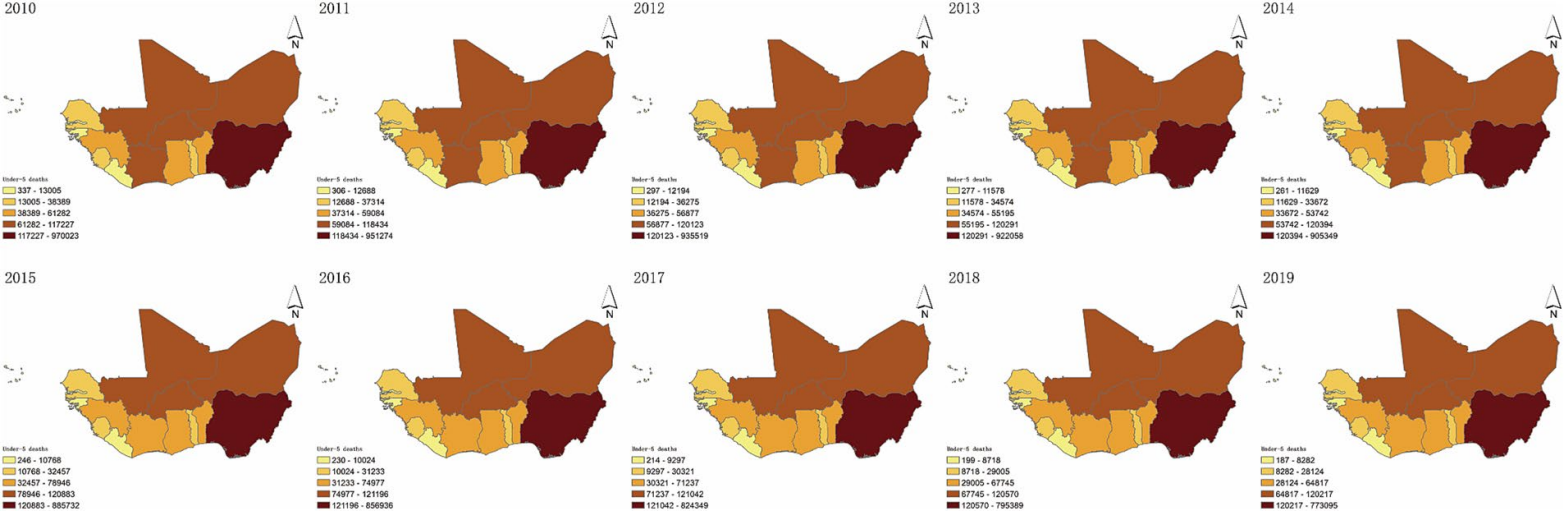
The spatial distribution of under-5 mortality rate from 2010 to 2019 in West African countries.

Concerning the geographical distribution, the spatial variations of U5MR during 2010–2019 are depicted in Figure 2. To evaluate the spatial distribution patterns of U5MR, the spatial autocorrelation at the country level was examined based on the global Moran’s *I* statistic and *Z* test (Table 3). The global Moran’s *I* statistics of U5MR were significant and greater than zero representing the positive spatial autocorrelation in the overall spatial distribution. Subsequently, the global Moran’s *I* statistics slightly increased from 0.2850 (*p* = 0.0210) to 0.3597 (*p* = 0.0080) during the study period, illustrating that the agglomeration trend of U5MR and differences within regions were gradually increased. Notably, we found the non-significant clustering of LISA coefficients of U5MR from 2010 to 2019 in West African countries.

**Table 3:** The global Moran’s *I* with value of *p*-values of under-5 mortality rate from 2010 to 2019 in West African countries.

Year	Moran’s *I*	*z* score	*p*-value
2010	0.2850	2.1722	0.0210^*^
2011	0.2991	2.2620	0.0190^*^
2012	0.3099	2.3237	0.0190^*^
2013	0.3276	2.4309	0.0150^*^
2014	0.3349	2.4802	0.0130^*^
2015	0.3428	2.5248	0.0100^*^
2016	0.3493	2.5610	0.0080^*^
2017	0.3541	2.5864	0.0080^**^
2018	0.3573	2.6046	0.0080^**^
2019	0.3597	2.6180	0.0080^**^

^*^p < 0.05, ^**^p < 0.01.

### 3.2. Association between health resources and services and U5MR

To explore the differential impacts of health resources and services on U5MR, we further constructed the OLS regression analyses (Supplementary Table S2), whose coefficients were not statistically significant. Further, the estimated results for the spatial regression models with LM diagnostics are presented in Table 4. This indicated that there was spatial dependence based on the above global spatial autocorrelation, requiring SEM and SLM analyses to be conducted. Thus, spatial regression models were more suitable than ordinary least square regression analysis in this study.

**Table 4:** The spatial dependence by Lagrange multiplier.

Year		LM (lag)	Robust LM (lag)	LM (error)	Robust LM (error)
2010 (DF = 1)	value	0.0880	0.0422	0.0478	0.0020
	*p*-value	0.7667	0.8371	0.8269	0.9642
2011 (DF = 1)	value	4.4619	4.7254	0.2887	0.5521
	*p*-value	0.0347^*^	0.0297^*^	0.5911	0.4574
2012 (DF = 1)	value	5.6591	6.3960	1.5239	2.2608
	*p*-value	0.0174^*^	0.0114^*^	0.2170	0.1327
2013 (DF = 1)	value	7.2158	9.3787	0.2488	2.4117
	*p*-value	0.0072^*^	0.0022^*^	0.6179	0.1204
2014 (DF = 1)	value	2.3800	7.0032	1.1115	5.7346
	*p*-value	0.1229	0.0081^*^	0.2918	0.0166^*^
2015 (DF = 1)	value	0.1097	0.6044	0.8839	1.3785
	*p*-value	0.7405	0.4369	0.3472	0.2404
2016 (DF = 1)	value	2.0982	11.1687	0.1178	9.1883
	*p*-value	0.1475	0.0008^*^	0.7314	0.8962
2017 (DF = 1)	value	4.5796	7.7113	1.5608	4.6925
	*p*-value	0.0323^*^	0.0055^*^	0.2116	0.0303^*^
2018 (DF = 1)	value	14.1569	14.6467	4.4580	4.9478
	*p*-value	0.0002^*^	0.0001^*^	0.0347^*^	0.0261^*^
2019 (DF = 1)	value	6.0226	7.8026	0.9370	2.7170
	*p*-value	0.0141^*^	0.0052^*^	0.3330	0.0992

LM, Lagrange multiplier; robust LM, robust Lagrange multiplier; DF: degree of freedom. ^*^p < 0.05.

The goodness-of-fit statistics, such as AICs, SCs, log-likelihoods, and *R*-squared, were used to estimate the degree of fit of the regression models (Table 5). The regression results from SEM, SLM, and OLS are listed in Table 6; Supplementary Tables S1, S2. The AIC of OLS was higher than that of SLM and SEM, and the AIC of SEM was lower than that of SLM. The *R*-squared of SEM was higher than that of OLS and SLM. Based on AIC, SC, and *R*-squared, SEM performed better than both the OLS and SLM with statistical significance for the spatial parameters. The spatial parameters of SEM were statistically significant, indicating that there was a significant spatial autocorrelation of U5MR in West African countries. Therefore, the SEM was adopted to explore the spatial effects of health resources and services on U5MR for further investigation.

**Table 5:** Comparison of goodness of fit of ordinary least square, spatial lag model and spatial error model.

Model	Statistic	2010	2011	2012	2013	2014	2015	2016	2017	2018	2019
Ordinary least square	AIC	−135.4660	−181.7190	−171.5520	−137.940	−126.6270	−136.2340	−121.7780	−115.3220	−120.9910	−126.1390
	SC	−126.2620	−172.5140	−162.3470	−128.7350	−117.4220	−127.0290	−112.5740	−106.1180	−111.7870	−116.9350
	Log likelihood	80.7332	103.8590	98.7758	81.9700	76.3135	81.1169	73.8892	70.6611	73.4957	76.0696
	*R*-squared	0.9832	0.9992	0.9984	0.9839	0.9637	0.9798	0.9435	0.9069	0.9322	0.9485
	Spatial parameter (*λ*)	—	—	—	—	—	—	—	—	—	—
	Moran’s *I* of residuals	−0.0342	−0.1549	−0.3051	−0.1008	−0.2034	−0.2252	−0.1148	−0.2371	−0.3279	−0.2246
Spatial lag model	AIC	−136.5300	−182.1290	−178.0850	−147.1980	−125.2260	−134.3780	−120.5570	−122.5230	−135.0780	−136.8070
	SC	−126.6170	−172.2160	−168.1720	−137.2850	−115.3130	−124.4650	−110.6440	−112.6100	−125.1660	−126.8950
	Log likelihood	82.2648	105.0640	103.0420	87.5988	76.6128	81.1890	74.2783	75.2613	81.5391	82.4037
	*R*-squared	0.9868	0.9993	0.9990	0.9928	0.9654	0.9800	0.9487	0.9428	0.9799	0.9670
	Spatial parameter (*λ*)	−0.4058^*^	0.0485	0.1189^*^	0.4597^**^	0.1829	−0.0778	−0.4347	−0.6111^*^	−0.9012^**^	−0.4649
	Moran’s *I* of residuals	−0.1085	−0.0190	−0.2070	−0.1473	−0.2529	−0.2157	−0.1123	−0.2393	−0.2496	−0.2151
Spatial error model	AIC	−148.1140	−195.4830	−186.0810	−142.8530	−136.5160	−150.5600	−129.9500	−126.3660	−133.6070	−139.0070
	SC	−138.909	−186.2780	−176.8760	−133.6480	−127.3120	−141.3560	−120.7450	−117.1610	−124.4030	−129.8030
	Log likelihood	87.0570	110.7414	106.0404	84.4264	81.2582	88.2802	77.9748	76.1828	79.8037	82.5036
	*R*-squared	0.9992	1.0000	1.0000	0.9997	0.9951	0.9991	0.9932	0.9956	0.9991	0.9998
	Spatial parameter (*λ*)	−1.4605^**^	−1.5564^**^	−1.9450^**^	−1.8011^**^	−1.2775^**^	−1.3611^**^	−1.4261^**^	−2.2813^**^	−1.7644^**^	−1.9924^**^
	Moran’s *I* of residuals	−0.5280^*^	−0.5672^*^	−0.5181^**^	−0.5245^*^	−0.5302^**^	−0.5956^*^	−0.5202^*^	−0.4166^*^	−0.5270^*^	−0.5000^*^

AIC, Akaike information criterion; SC, Schwartz criterion. ^*^p < 0.05, ^**^p < 0.01.

**Table 6:** The association between health resources and services and under-5 mortality rate in West African countries, 2010–2019: estimated from spatial error model.

Independent variables	2010		2011		2012		2013		2014	
	Coefficient	*p*-value	Coefficient	*p*-value	Coefficient	*p*-value	Coefficient	*p*-value	Coefficient	*p*-value
HDI	−0.0752	<0.0001^**^	−0.1054	<0.0001^**^	−0.0898	<0.0001^**^	−0.0187	<0.0001^**^	−0.0613	<0.0001^**^
Phy	−0.0086	<0.0001^**^	−0.0029	<0.0001^**^	−0.0056	<0.0001^**^	−0.0049	<0.0001^**^	0.0028	<0.0001^**^
Nam	−0.0019	<0.0001^**^	−0.0012	<0.0001^**^	−0.0010	<0.0001^**^	−0.0021	<0.0001^**^	−0.0035	<0.0001^**^
Php	0.0019	<0.0001^**^	0.0016	<0.0001^**^	0.0002	<0.0001^**^	−0.0003	0.1985	0.0095	<0.0001^**^
Hc	0.0001	<0.0001^**^	0.0002	<0.0001^**^	0.0004	<0.0001^**^	0.0005	<0.0001^**^	−0.0004	<0.0001^**^
CHE	0.0006	<0.0001^**^	0.0003	<0.0001^**^	0.0004	<0.0001^**^	0.0003	<0.0001^**^	0.0000	0.0716
Dtp	−0.0003	0.00001^*^	0.0002	<0.0001^**^	0.0005	<0.0001^**^	0.0008	<0.0001^**^	0.0000	0.6564
Mea	−0.0002	<0.0001^**^	−0.0002	<0.0001^**^	−0.0003	<0.0001^**^	−0.0007	<0.0001^**^	−0.0007	<0.0001^**^
Br	−0.0001	0.0002^*^	0.0000	<0.0001^**^	0.0001	<0.0001^**^	0.0001	<0.0001^**^	−0.0003	<0.0001^**^
Ant	−0.0010	<0.0001^**^	−0.0006	<0.0001^**^	−0.0003	<0.0001^**^	−0.0003	<0.0001^**^	−0.0010	<0.0001^**^
Con	−0.0001	0.0040^**^	0.0000	<0.0001^**^	−0.0001	<0.0001^**^	0.0001	<0.0001^**^	0.0000	0.4284
Shp	0.0001	0.0226^*^	0.0003	<0.0001^**^	0.0001	<0.0001^**^	0.0000	0.4981	0.0003	<0.0001^**^

Independent variables	2015		2016		2017		2018		2019	
	Coefficient	*p*-value	Coefficient	*p*-value	Coefficient	*p*-value	Coefficient	*p*-value	Coefficient	*p*-value
HDI	−0.0609	<0.0001^**^	−0.0973	<0.0001^**^	−0.1323	<0.0001^**^	−0.0423	<0.0001^**^	−0.0795	<0.0001^**^
Phy	0.0016	<0.0001^**^	0.0025	<0.0001^**^	0.0012	0.2540	0.0058	<0.0001^**^	0.0055	<0.0001^**^
Nam	−0.0022	<0.0001^**^	−0.0017	<0.0001^**^	−0.0028	<0.0001^**^	0.0002	0.0761	−0.0001	0.0089^**^
Php	0.0087	<0.0001^**^	0.0126	<0.0001^**^	0.0203	<0.0001^**^	0.0037	<0.0001^**^	0.0080	<0.0001^**^
Hc	0.0000	0.1007	0.0000	0.3732	−0.0003	0.0013^**^	0.0001	0.0006^*^	0.0002	<0.0001^**^
CHE	0.0000	<0.0001^**^	0.0000	0.5542	0.0003	0.0871	−0.0006	<0.0001^**^	−0.0005	<0.0001^**^
Dtp	0.0006	<0.0001^**^	0.0009	<0.0001^**^	0.0021	<0.0001^**^	−0.0002	0.1027	0.0005	<0.0001^**^
Mea	−0.0008	<0.0001^**^	−0.0009	<0.0001^**^	−0.0023	<0.0001^**^	0.0002	0.0879	−0.0002	<0.0001^**^
Br	0.0000	0.6109	−0.0002	<0.0001^**^	−0.0001	0.0394^*^	0.0000	0.0439^*^	0.0002	<0.0001^**^
Ant	−0.0005	<0.0001^**^	−0.0011	<0.0001^**^	−0.0010	<0.0001^**^	−0.0001	0.1475	0.0000	0.0162^*^
Con	−0.0001	<0.0001^**^	0.0001	0.1263	−0.0007	0.0292^*^	0.0006	<0.0001^**^	0.0005	<0.0001^**^
Shp	0.0001	<0.0001^**^	0.0005	<0.0001^**^	0.0005	0.0006^*^	−0.0001	0.0397^*^	−0.0002	<0.0001^**^

^*^p < 0.05, ^**^p < 0.01.

The results of SEM (Table 6) revealed that HDI, physicians density, nurses and midwives density, health center density, percentage of infants lacking immunization for diphtheria and measles, and coverage rate of at least one antenatal visit were negatively and significantly correlated with U5MR at country level in West Africa. Conversely, Current health expenditure *per capita* was a significantly positive influence on U5MR. In addition, pharmaceutical personnel density, birth registration rate of children under 5 years of age, contraceptive rate of women of reproductive age 15–49 years, and proportion of births attended by skilled health personnel showed a slight influence on U5MR from SEM.

To ensure the accuracy of the study results, the sensitivity analysis was performed by transforming the “Queen contiguity” matrix to “Rook adjacency” matrix, and the results were similar to the results above (Supplementary material).

## 4. Discussion

Our study examined spatiotemporal trends and impacts of health resources and services by spatial spillover effects during the study period. Overall, U5MR decreased at different speeds, with the AAPCs and APCs being statistically significant in 15 West African countries. The most significant decrease in U5MR occurred in 2013, 2014, and 2015, with several decreased APCs during 2015–2019 being higher than APCs during 2010–2019. Spatial autocorrelation analysis revealed that the global Moran’s *I* statistic of U5MR from 2010 to 2019 was greater than zero and statistically significant, indicating that global spatial clustering was observed in adjacent countries. However, the local spatial autocorrelation was not statistically significant. The global Moran’s *I* statistic increased in the study period, indicating the global spatial autocorrelation and clustering trend of U5MR in adjacent countries and regional variations gradually increased. In addition, we explored spatial spillover effects of health resources and services on U5MR in West African countries. Increasing HDI, physicians density, nurses and midwives density, health center density, percentage of infants lacking immunization for diphtheria and measles, and coverage rate of at least one antenatal visit can reduce U5MR in their own and adjacent countries. Meanwhile, a positive correlation was observed between Current health expenditure *per capita* and U5MR through positive spatial spillover effects.

The U5MR was observed in a long-term downward trend, and significant progress was made in reducing U5MR in West African countries. Since 2000, West African countries signed the United Nations Millennium Development Goal of “reducing the U5MR by two-thirds by 2015,” and the United Nations proposed SDG of “reducing the U5MR to at least 25 deaths per 1,000 live births in all countries by 2030” (28). Specifically, the 2014–2016 Ebola outbreak in West Africa, particularly in Guinea, Liberia (29), Senegal, Sierra Leone (30), Mali, Nigeria, and Senegal (31), led to trends in child mortality. Weak health systems in West African countries have been less resilient to the impact of the Ebola outbreak, contributing to an increase in U5MR (32). Furthermore, our study analyzed the spatial autocorrelation of U5MR. We showed that U5MR presented positive spatial autocorrelation during our study period from the spatiotemporal perspective. The U5MR of the local country was influenced by the U5MR of adjacent countries; the higher the U5MR of adjacent countries, the higher the U5MR of the local country. Few studies have aligned with the previous correlation analysis of inequity on U5MR by distribution (33, 34). However, in this study, we interpreted the existing positive spatial autocorrelation to indicate the inequity; in other words, we identified regional similarity and clustering. Our study also revealed that spatial autocorrelation increased from 2010 to 2019, suggesting that regional clustering on U5MR increased. Recently, relevant United Nations agencies and charities have implemented multi-faceted global health governance, such as the United Nations Population Fund, UNICEF, the World Health Organization, the Joint United Nations Program on human immunodeficiency virus/acquired immunodeficiency syndrome, the World Bank, the Bill and Melinda Gates Foundation, etc., to help Africa reduce the gap of U5MR.

The recent study indicated that efforts to unmask under-5 mortality patterns through several lenses, including high spatial and temporal resolution, socioeconomic decomposition (6, 35, 36). Socioeconomic development has played a key role in improving children’s health. Consistent with the previous ecological study, the HDI was negatively correlated with U5MR (15). From the spatial perspective, the present study demonstrated that the HDI was negatively correlated with U5MR, and the correlation increased through the spatial spillover effects. Interactions from spatial neighborhoods between HDI and U5MR might be explained by the increase in local socioeconomic development, which might decrease the local and adjacent mortality rate. In terms of regional socioeconomic development, as the largest regional economic organization in Africa, the ECOWAS should consider the spatial spillover effects of the socioeconomic development strategy, strengthen cooperation among neighboring countries, and jointly promote regional economy to reduce U5MR.

Adequate and equitably distributed health resources have been critical to improving population health and achieving universal health coverage and SDGs (37–39). The U5MR of West African countries was not only related to the level of their own health human resources but also related to the U5MR of adjacent countries. This finding was consistent with a previous study, which found that in developed countries, a relatively high density of nurses and midwives was associated with lower U5MR (40). However, the level and distribution of health human resources in West African countries have always been factors hindering the development of population health, which not only affected the health level of children in their local countries but also affected the health level of children in adjacent countries. African countries faced enormous challenges in terms of education, personnel training, and quantity and regional distribution of health workers, especially the acute shortage of physicians, nurses, and midwives (41, 42). In 2015, only 11 African countries met the World Health Organization’s minimum density of 22.8 per 100,000 population health workers (including physicians, nurses, and midwives) (43). In 2018, the density of physicians, nurses, and midwives in the World Health Organization African Region was 1.55 per 100,000 population. Only Seychelles, Namibia, Mauritius, and South Africa have made progress in universal health coverage, and the density of physicians, nurses, and midwives exceeded 4.45 per 100,000 people, but not in West African countries (44). In addition to strengthening the training of health manpower, West African countries need to grasp the differences between different countries in formulating health manpower regulation policies to improve children’s health to a certain extent. Previous studies have demonstrated that increasing health expenditure can reduce U5MR, and similar results have been found in East African countries (45). Additionally, Olatunde et al. used a fully modified least squares panel model to determine the long-term negative correlation between current health expenditure (CHE) and U5MR in West African countries from 1991 to 2015 (46). Similar results were found in single-country studies in Nigeria and Ghana, where increased government spending on health significantly improved child mortality (47, 48). However, the above studies did not consider spatial correlation, and the present study filled this gap; per CHE had negative spatial spillover effect on U5MR. In addition to being associated with U5MR in one country, health expenditure may also affect U5MR in adjacent countries.

To achieve the sustainable goal of U5MR, the quality and equity of antenatal care and routine vaccination should be paid attention to. The health services of adjacent countries might affect children’s health in their local countries to some extent. Although the Expanded Program on Immunization was launched by the World Health Organization in 1974, it has facilitated coordinated progress at the national level in routine vaccination (such as diphtheria, tetanus, pertussis, and measles). However, routine vaccination rates in West African countries were still the lowest, accelerating the spread of infectious diseases in adjacent countries, thereby increasing the burden of infectious diseases and U5MR (49). In addition, antenatal care and skilled delivery became the guarantee of maternal and child health, and unequal distribution of antenatal care coverage in West African countries may also increase the U5MR in neighboring countries (50). Therefore, West African countries should make full use of the regional spillover effects mechanism and strengthen medical exchanges and cooperation with neighboring countries to realize the effective supply of local basic medical services.

In summary, West African countries experienced the highest U5MR and the lowest life expectancy globally. Few studies have explored the correlation between health resources and services and U5MR from the spatiotemporal perspective. However, this study had several limitations. First, this study used panel data and cannot infer cause and effect, but it was combined with geographic information systems to provide a more comprehensive understanding of the spatiotemporal characteristics of U5MR and its spatial spillover effects on health resources and services in West African countries. Second, our study was based on secondary databases and might have certain data quality limitations. However, we selected three globally recognized and authoritative databases, the World Health Organization Global Health Observatory, the United Nations Human Development Report, and the HIME Global Burden of Disease Study, to increase the reliability of data. Third, the indicators of health resources and services may not capture the comprehensive interplay of their process toward U5MR; hence, missing variables and potential confounders, such as unsafe drinking water and poor sanitation facilities, were inevitable and may have left some spatial spillover effects unexplained. Unsafe drinking water and poor sanitation facilities might increase U5MR through the spread of infectious diseases (51). Finally, our study did not consider the prediction of the spatiotemporal trend of U5MR in West African countries. To address these shortcomings, establishing the prediction model of influencing factors of U5MR in West African countries from the spatiotemporal perspective should be considered in order to promote regional health and global health development in the future.

## 5. Conclusion

We found geographical variations with global spatial clustering of U5MR at country level in West Africa during 2010–2019. Under the spatiotemporal trend analysis, we identified that regional clustering and regional variations on U5MR gradually increased. Meanwhile, based on exploratory spatial data analysis, we interpreted how health resources and services proxies influence U5MR through spatial spillover effects. Increasing the level of economic development and health human resources (especially nurses and midwives), routine vaccine coverage (diphtheria and measles vaccines), antenatal care coverage, and the proportion of births attended by health professionals may not only reduce U5MR in the local country but also in adjacent countries. We suggest that when making regional health policy and intervention cooperation mechanisms, it is necessary for West African regional organizations, such as the ECOWAS and its affiliated organization and the West African Health Organization, to consider the spatial spillover effects between adjacent countries, which is of great significance for improving children’s health, achieving the 2030 SDGs, Africa Agenda 2063, and universal health coverage.

## Data availability statement

The datasets presented in this study can be found in online repositories. The names of the repository/repositories and accession number(s) can be found at: https://www.healthdata.org/gbd/2019; https://www.who.int/data/gho; https://hdr.undp.org.

## Ethics statement

The article does not contain any studies with human participants or animals performed by any of the authors. Ethical review and approval was not required because this study disclosed no personal information and used public data freely shared by the Global Burden of Disease (GBD) database (https://www.healthdata.org/about/data), the World Health Organization Global Health Observatory (https://www.who.int/data/gho), and the United Nations Human Development Report (https://hdr.undp.org).

## Author contributions

MZ and LN designed the study. MZ performed statistical analyses and drafted the manuscript. LN obtained funding and revised the manuscript. All authors contributed to the article and approved the submitted version.

## Funding

This research was funded Philosophical and Social Science Fund of Hunan Province, China (20YBQ05). The funders played no role in the study design, data collection, data analysis, data interpretation, or writing of the report.

## Conflict of interest

The authors declare that the research was conducted in the absence of any commercial or financial relationships that could be construed as a potential conflict of interest.

## Publisher’s note

All claims expressed in this article are solely those of the authors and do not necessarily represent those of their affiliated organizations, or those of the publisher, the editors and the reviewers. Any product that may be evaluated in this article, or claim that may be made by its manufacturer, is not guaranteed or endorsed by the publisher.

## Footnotes

^1^ https://hdr.undp.org/

^2^ https://surveillance.cancer.gov/joinpoint/
