# Supplementary material for: Exploring spatiotemporal trends and impacts of health resources and services on under-5 mortality in West African countries, 2010–2019: a spatial data analysis
Source: Front Public Health. 2023 Sep 13;11:1193319. doi: 10.3389/fpubh.2023.1193319 (PMC10524609; doi:10.3389/fpubh.2023.1193319)
Supplement: Supplementary file 1 [file Data_Sheet_1.docx]

Supplementary Material

Exploring Spatiotemporal Trends and Impacts of Health Resources and Services on Under-5 Mortality in West African Countries, 2010-2019: A Spatial Data Analysis

**Meng Zeng**^1^**, Lu Niu**^1^**^*^**

^1^ Department of Social Medicine and Health Management, Xiangya School of Public Health, Central South University, No. 238, Shang Ma Yuan ling Alley, Kaifu District, Changsha, Hunan, 410078, China.

**^*^Correspondence:**

Corresponding Author: Lu Niu.

Department of Social Medicine and Health Management, Xiangya School of Public Health, Central South University, No. 238, Shang Ma Yuan ling Alley, Kaifu District, Changsha, Hunan, 410078, China.

**E-mail:** [**niu_lu@csu.edu.cn**](mailto:niu_lu@csu.edu.cn)

# Supplementary Figures and Tables

## Supplementary Figures


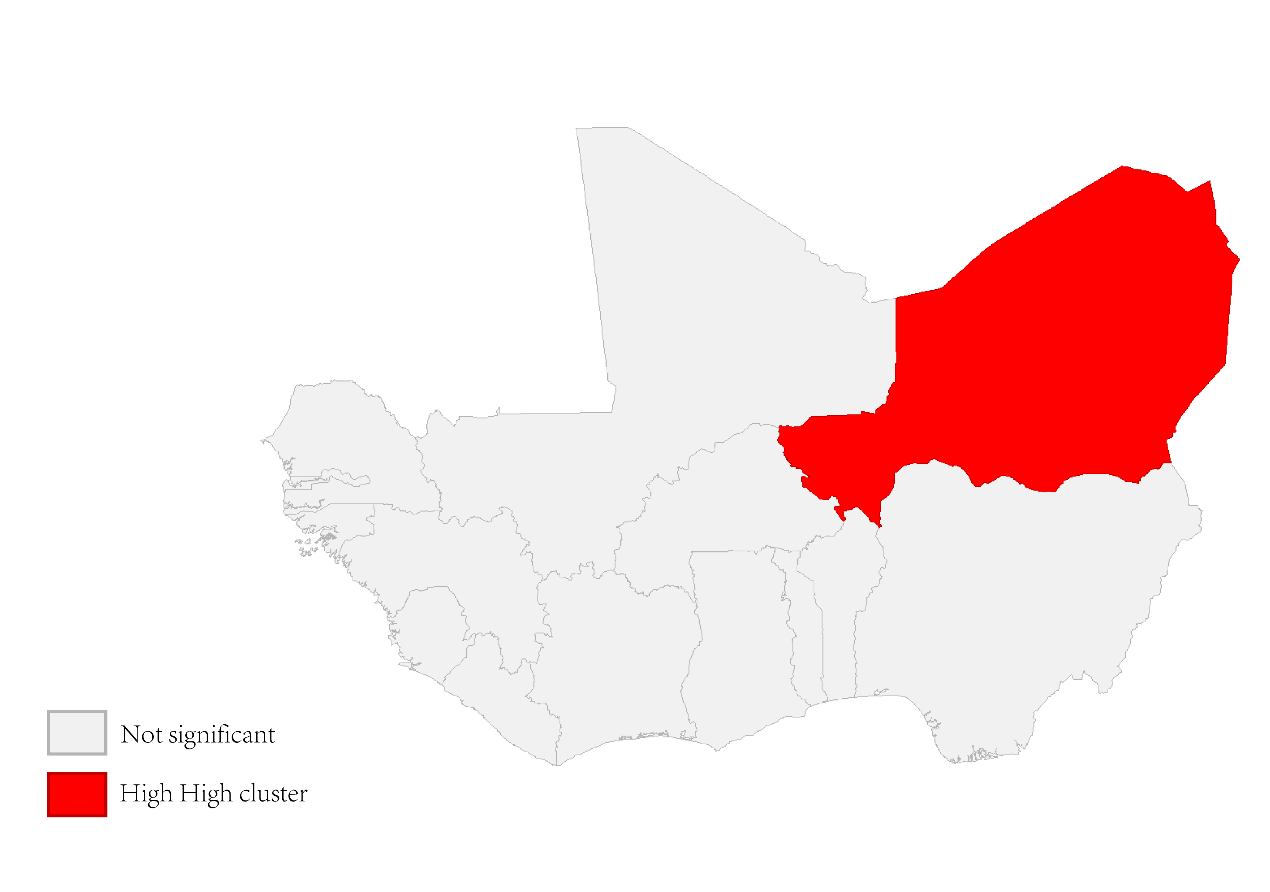


**Supplementary Figure 1.** The spatial variation of LISA coefficients of under-5 mortality rate in West African countries, 2010-2019 in sensitive analysis.

## Supplementary Tables

TABLE S1 The association between health resources and services and under-5 mortality rate in West African countries, 2010-2019: estimated from spatial lag model.

| Independent variables | 2010 | | 2011 | | 2012 | | 2013 | | 2014 | |
| --- | --- | --- | --- | --- | --- | --- | --- | --- | --- | --- |
|  | coefficient | p-value | coefficient | p-value | coefficient | p-value | coefficient | p-value | coefficient | p-value |
| HDI | -0.0925 | <0.0001^**^ | -0.1048 | <0.0001^**^ | -0.0892 | <0.0001^**^ | -0.0556 | <0.0001^**^ | -0.0811 | <0.0001^**^ |
| Phy | -0.0047 | 0.0033^**^ | -0.0027 | <0.0001^**^ | -0.0051 | <0.0001^**^ | -0.0042 | <0.0001^**^ | 0.0014 | 0.0810 |
| Nam | -0.0012 | 0.0001^**^ | -0.0012 | <0.0001^**^ | -0.0010 | <0.0001^**^ | -0.0011 | <0.0001^**^ | -0.0018 | <0.0001^**^ |
| Php | 0.0029 | 0.0001^**^ | 0.0016 | <0.0001^**^ | 0.0003 | 0.1395 | -0.0004 | 0.4481 | 0.0064 | <0.0001^**^ |
| Hc | 0.0003 | <0.0001^**^ | 0.0002 | <0.0001^**^ | 0.0004 | <0.0001^**^ | 0.0003 | <0.0001^**^ | 0.0000 | 0.6945 |
| CHE | 0.0003 | 0.0023^**^ | 0.0003 | <0.0001^**^ | 0.0003 | <0.0001^**^ | 0.0003 | <0.0001^**^ | 0.0000 | 0.0200^*^ |
| Dtp | 0.0002 | 0.1745 | 0.0003 | <0.0001^**^ | 0.0006 | <0.0001^**^ | 0.0006 | <0.0001^**^ | 0.0002 | 0.0194^*^ |
| Mea | -0.0002 | 0.0020^**^ | -0.0002 | <0.0001^**^ | -0.0003 | <0.0001^**^ | -0.0004 | <0.0001^**^ | -0.0004 | 0.0007^**^ |
| Br | 0.0000 | 0.9313 | -0.0001 | 0.0001^**^ | 0.0001 | <0.0001^**^ | 0.0000 | 0.1811 | -0.0002 | 0.0335 |
| Ant | -0.0007 | 0.0001^**^ | -0.0007 | <0.0001^**^ | -0.0003 | <0.0001^**^ | -0.0004 | <0.0001^**^ | -0.0007 | 0.0001^**^ |
| Con | -0.0001 | 0.3627 | -0.0001 | 0.0001^**^ | -0.0001 | <0.0001^**^ | -0.0001 | 0.0012^**^ | 0.0000 | 0.5611 |
| Shp | 0.0002 | 0.0126^*^ | 0.0003 | <0.0001^**^ | 0.0002 | <0.0001^**^ | 0.0003 | <0.0001^**^ | 0.0003 | 0.0023^**^ |

^*^p<0.05; ^**^p<0.01.

Continued:

| Independent variables | 2015 | | 2016 | | 2017 | | 2018 | | 2019 | |
| --- | --- | --- | --- | --- | --- | --- | --- | --- | --- | --- |
|  | coefficient | p-value | coefficient | p-value | coefficient | p-value | coefficient | p-value | coefficient | p-value |
| HDI | -0.0835 | <0.0001^**^ | -0.0978 | <0.0001^**^ | -0.0960 | 0.0001^**^ | -0.0615 | <0.0001^**^ | -0.0704 | <0.0001^**^ |
| Phy | 0.0006 | 0.1454 | 0.0006 | 0.3807 | 0.0024 | 0.1561 | 0.0041 | <0.0001^**^ | 0.0036 | 0.0016^**^ |
| Nam | -0.0015 | <0.0001^**^ | -0.0011 | 0.0010^**^ | -0.0009 | 0.0640 | -0.0004 | 0.0012^**^ | -0.0003 | 0.0090^**^ |
| Php | 0.0071 | <0.0001^**^ | 0.0080 | <0.0001^**^ | 0.0090 | 0.0033^**^ | 0.0068 | <0.0001^**^ | 0.0070 | <0.0001^**^ |
| Hc | 0.0001 | 0.0595 | 0.0000 | 0.7163 | 0.0001 | 0.6248 | 0.0001 | 0.0022^**^ | 0.0002 | 0.0008^**^ |
| CHE | 0.0000 | 0.0142^*^ | 0.0000 | 0.7857 | -0.0002 | 0.3323 | -0.0004 | <0.0001^**^ | -0.0004 | 0.0011^**^ |
| Dtp | 0.0006 | <0.0001^**^ | 0.0004 | 0.0005^**^ | 0.0004 | 0.4065 | 0.0002 | 0.1377 | 0.0004 | <0.0001^**^ |
| Mea | -0.0005 | <0.0001^**^ | -0.0005 | 0.0103^*^ | -0.0005 | 0.3663 | -0.0003 | 0.0191^**^ | -0.0002 | 0.0204^*^ |
| Br | 0.0000 | 0.7360 | 0.0000 | 0.9767 | 0.0001 | 0.2176 | 0.0002 | <0.0001^**^ | 0.0002 | <0.0001^**^ |
| Ant | -0.0003 | 0.0039^**^ | -0.0006 | 0.0009^**^ | -0.0005 | 0.0094^**^ | -0.0003 | 0.0088^**^ | 0.0000 | 0.9605 |
| Con | 0.0001 | 0.1927 | 0.0002 | 0.0648 | 0.0004 | 0.2500 | 0.0006 | <0.0001^**^ | 0.0006 | 0.0001^**^ |
| Shp | 0.0002 | 0.0017^**^ | 0.0001 | 0.4009 | -0.0001 | 0.6500 | -0.0003 | <0.0001^**^ | -0.0003 | 0.0041^**^ |

^*^p<0.05; ^**^p<0.01.

TABLE S2 The association between health resources and services and under-5 mortality rate in West African countries, 2010-2019: estimated from ordinary least square.

| Independent variables | 2010 | | 2011 | | 2012 | | 2013 | | 2014 | |
| --- | --- | --- | --- | --- | --- | --- | --- | --- | --- | --- |
|  | coefficient | p-value | coefficient | p-value | coefficient | p-value | coefficient | p-value | coefficient | p-value |
| HDI | -0.0891 | 0.1468 | -0.1053 | 0.0057 | -0.0941 | 0.0117 | -0.0806 | 0.1431 | -0.0880 | 0.1485 |
| Phy | -0.0053 | 0.3877 | -0.0028 | 0.0560 | -0.0048 | 0.0329 | -0.0031 | 0.2848 | 0.0014 | 0.6055 |
| Nam | -0.0017 | 0.1113 | -0.0012 | 0.0079 | -0.0010 | 0.0207 | -0.0011 | 0.2095 | -0.0018 | 0.2668 |
| Php | 0.0034 | 0.2697 | 0.0016 | 0.1314 | 0.0007 | 0.4165 | 0.0011 | 0.6202 | 0.0066 | 0.1951 |
| Hc | 0.0002 | 0.2578 | 0.0002 | 0.0155 | 0.0004 | 0.0161 | 0.0002 | 0.2063 | 0.0000 | 0.8528 |
| CHE | 0.0004 | 0.2599 | 0.0003 | 0.0123 | 0.0003 | 0.0149 | 0.0002 | 0.1117 | 0.0000 | 0.5255 |
| Dtp | -0.0001 | 0.8676 | 0.0003 | 0.0644 | 0.0005 | 0.0226 | 0.0004 | 0.2329 | 0.0002 | 0.5218 |
| Mea | -0.0002 | 0.3092 | -0.0002 | 0.0357 | -0.0003 | 0.0861 | -0.0003 | 0.3823 | -0.0004 | 0.3502 |
| Br | -0.0001 | 0.4079 | 0.0000 | 0.3373 | 0.0001 | 0.1583 | 0.0000 | 0.7960 | -0.0002 | 0.5365 |
| Ant | -0.0010 | 0.1287 | -0.0007 | 0.0098 | -0.0003 | 0.1120 | -0.0005 | 0.2732 | -0.0007 | 0.2609 |
| Con | -0.0001 | 0.6968 | 0.0000 | 0.3484 | -0.0001 | 0.2076 | -0.0001 | 0.7495 | 0.0001 | 0.7836 |
| Shp | 0.0003 | 0.2298 | 0.0003 | 0.0207 | 0.0002 | 0.0871 | 0.0002 | 0.2647 | 0.0003 | 0.4037 |

Continued:

| Independent  variables | 2015 | | 2016 | | 2017 | | 2018 | | 2019 | |
| --- | --- | --- | --- | --- | --- | --- | --- | --- | --- | --- |
|  | coefficient | p-value | coefficient | p-value | coefficient | p-value | coefficient | p-value | coefficient | p-value |
| HDI | -0.0818 | 0.1000 | -0.0937 | 0.1733 | -0.0973 | 0.3636 | -0.0629 | 0.4708 | -0.0689 | 0.2929 |
| Phy | 0.0006 | 0.6616 | 0.0003 | 0.9050 | 0.0010 | 0.8703 | 0.0035 | 0.5104 | 0.0023 | 0.5429 |
| Nam | -0.0015 | 0.1480 | -0.0009 | 0.4176 | -0.0008 | 0.6808 | -0.0003 | 0.6401 | -0.0004 | 0.4460 |
| Php | 0.0068 | 0.1019 | 0.0061 | 0.2493 | 0.0075 | 0.5478 | 0.0050 | 0.3369 | 0.0060 | 0.1193 |
| Hc | 0.0001 | 0.4990 | 0.0001 | 0.7560 | 0.0000 | 0.9093 | 0.0002 | 0.5091 | 0.0002 | 0.4785 |
| CHE | 0.0000 | 0.3866 | 0.0000 | 0.8350 | -0.0001 | 0.9480 | -0.0003 | 0.5546 | -0.0003 | 0.4902 |
| Dtp | 0.0006 | 0.1332 | 0.0004 | 0.3317 | 0.0005 | 0.7750 | 0.0002 | 0.7659 | 0.0005 | 0.2245 |
| Mea | -0.0005 | 0.1952 | -0.0003 | 0.5461 | -0.0004 | 0.8301 | -0.0001 | 0.8535 | -0.0003 | 0.4856 |
| Br | 0.0000 | 0.9259 | 0.0000 | 0.9590 | 0.0001 | 0.7754 | 0.0001 | 0.5103 | 0.0002 | 0.3266 |
| Ant | -0.0003 | 0.3896 | -0.0004 | 0.4327 | -0.0003 | 0.6822 | -0.0001 | 0.8386 | 0.0000 | 0.9660 |
| Con | 0.0001 | 0.6924 | 0.0001 | 0.6582 | 0.0002 | 0.8679 | 0.0005 | 0.4968 | 0.0005 | 0.3781 |
| Shp | 0.0002 | 0.3622 | 0.0001 | 0.6995 | 0.0000 | 0.9668 | -0.0001 | 0.7151 | -0.0002 | 0.5583 |

TABLE S3 The global Moran's I with p-values of under-5 mortality rate from 2010 to 2019 in West African countries in sensitive analysis.

| Year | Moran's I | z score | p-value |
| --- | --- | --- | --- |
| 2010 | 0.2209 | 1.6901 | 0.0490^*^ |
| 2011 | 0.2398 | 1.7938 | 0.0360^*^ |
| 2012 | 0.2565 | 1.8785 | 0.0340^*^ |
| 2013 | 0.2668 | 1.9301 | 0.0310^*^ |
| 2014 | 0.2850 | 2.0351 | 0.0250^*^ |
| 2015 | 0.2863 | 2.0361 | 0.0250^*^ |
| 2016 | 0.2871 | 2.0332 | 0.0240^*^ |
| 2017 | 0.2827 | 2.0026 | 0.0260^*^ |
| 2018 | 0.2819 | 1.9977 | 0.0250^*^ |
| 2019 | 0.2801 | 1.9865 | 0.0260^*^ |

^*^p<0.05.

TABLE S4 The spatial dependence by Lagrange multiplier in sensitive analysis.

| Year | | LM(lag) | Robust LM(lag) | LM(error) | | Robust LM(error) | |
| --- | --- | --- | --- | --- | --- | --- | --- |
| 2010  (DF=1) | value | 0.5578 | 8.1638 | 0.0650 | 7.6709 | |  |
|  | p-value | 0.4551 | 0.0043^*^ | 0.7988 | 0.0056^*^ | |  |
| 2011  (DF=1) | value | 1.9765 | 2.1145 | 0.0024 | 0.1404 | |  |
|  | p-value | 0.1598 | 0.1459 | 0.9611 | 0.7079 | |  |
| 2012  (DF=1) | value | 1.7486 | 2.4859 | 2.3969 | 3.1341 | |  |
|  | p-value | 0.1861 | 0.1149 | 0.1216 | 0.0767 | |  |
| 2013  (DF=1) | value | 11.8500 | 13.8221 | 0.0395 | 2.0117 | |  |
|  | p-value | 0.0006^*^ | 0.0002^*^ | 0.8424 | 0.1561 | |  |
| 2014  (DF=1) | value | 6.1074 | 13.5000 | 0.0868 | 7.4793 | |  |
|  | p-value | 0.0135 | 0.0002^*^ | 0.7684 | 0.0062^*^ | |  |
| 2015  (DF=1) | value | 4.5159 | 6.9918 | 0.1549 | 2.6309 | |  |
|  | p-value | 0.0336^*^ | 0.0082^*^ | 0.6939 | 0.1048 | |  |
| 2016  (DF=1) | value | 1.4980 | 7.9305 | 0.1553 | 6.5878 | |  |
|  | p-value | 0.2230 | 0.0049^*^ | 0.6935 | 0.0103^*^ | |  |
| 2017  (DF=1) | value | 1.0336 | 4.6733 | 0.0391 | 3.6788 | |  |
|  | p-value | 0.3093 | 0.0306^*^ | 0.8433 | 0.0551 | |  |
| 2018  (DF=1) | value | 0.0161 | 7.4847 | 0.0799 | 7.5484 | |  |
|  | p-value | 0.8990 | 0.0062^*^ | 0.7774 | 0.0060^*^ | |  |
| 2019  (DF=1) | value | 0.2853 | 1.4443 | 0.0001 | 1.1591 | |  |
|  | p-value | 0.5933 | 0.2294 | 0.9944 | 0.2817 | |  |

LM: Lagrange multiplier. Robust LM: Robust Lagrange multiplier. DF: degree of freedom. ^*^p<0.05.

TABLE S5 Comparison of goodness of fit of ordinary least square, spatial lag model and spatial error model in sensitive analysis.

| Model | Statistic | 2010 | 2011 | 2012 | 2013 | 2014 | 2015 | 2016 | 2017 | 2018 | 2019 |
| --- | --- | --- | --- | --- | --- | --- | --- | --- | --- | --- | --- |
| Ordinary least square | AIC | -135.4660 | -181.7190 | -171.5520 | -137.940 | -126.6270 | -136.2340 | -121.7780 | -115.3220 | -120.9910 | -126.1390 |
|  | SC | -126.2620 | -172.5140 | -162.3470 | -128.7350 | -117.4220 | -127.0290 | -112.5740 | -106.1180 | -111.7870 | -116.9350 |
|  | Log likelihood | 80.7332 | 103.8590 | 98.7758 | 81.9700 | 76.3135 | 81.1169 | 73.8892 | 70.6611 | 73.4957 | 76.0696 |
|  | R-squared | 0.9832 | 0.9992 | 0.9984 | 0.9839 | 0.9637 | 0.9798 | 0.9435 | 0.9069 | 0.9322 | 0.9485 |
|  | Spatial parameter(λ） | - | - | - | - | - | - | - | - | - | - |
|  | Moran's I of residuals | -0.0528 | -0.0103 | -0.3055 | -0.3099 | -0.0617 | -0.0796 | -0.0765 | -0.0380 | -0.0544 | 0.0005 |
| Spatial lag model | AIC | -134.6620 | -182.1440 | -171.5370 | -192.3110 | -145.5210 | -141.8080 | -122.4510 | -115.3420 | -119.0220 | -124.8120 |
|  | SC | -124.7500 | -172.2320 | -161.6240 | -182.3980 | -135.6080 | -131.8950 | -112.5380 | -105.4290 | -109.1090 | -114.9000 |
|  | Log likelihood | 81.3312 | 105.0720 | 99.76830 | 110.1550 | 86.7604 | 84.9038 | 75.2256 | 71.6709 | 73.5110 | 76.4061 |
|  | R-squared | 0.9854 | 0.9993 | 0.9986 | 0.9997 | 0.9942 | 0.9885 | 0.9578 | 0.9256 | 0.9324 | 0.9525 |
|  | Spatial parameter(λ） | 0.4572 | 0.1207 | 0.0722 | 0.6159^**^ | 0.9295^**^ | 0.4521^**^ | 0.5925^**^ | 0.5380^*^ | -0.0809 | 0.3571 |
|  | Moran's I of residuals | -0.0492 | 0.1193 | -0.2068 | -0.1895 | -0.1682 | -0.1581 | 0.0278 | 0.0553 | 0.0584 | 0.0187 |
| Spatial error model | AIC | -147.0710 | -187.9560 | -182.0730 | -139.6960 | -133.3880 | -143.6790 | -125.6630 | -125.6630 | -118.7390 | -134.8760 |
|  | SC | -137.8670 | -178.7510 | -172.8680 | -130.4920 | -124.1840 | -134.4740 | -116.4580 | -116.4580 | -109.5340 | -125.6710 |
|  | Log likelihood | 86.5356 | 106.9780 | 104.0365 | 82.8481 | 79.6941 | 84.8393 | 75.8315 | 75.8315 | 72.3695 | 80.4378 |
|  | R-squared | 0.9993 | 1.0000 | 0.9580 | 0.9991 | 0.9966 | 0.9976 | 0.9882 | 0.9883 | 0.9936 | 0.9864 |
|  | Spatial parameter(λ） | -1.4704^**^ | -1.6674^**^ | -15.9585^**^ | -1.8011^**^ | -1.6539^**^ | -1.3994^**^ | -2.6182^**^ | -2.6183^**^ | -1.7233^**^ | 0.9853^**^ |
|  | Moran's I of residuals | -0.4760^*^ | -0.4408 | -0.1473 | -0.4922^*^ | -0.5068^*^ | -0.4524^*^ | -0.4713^*^ | -0.4862^*^ | 0.5144^*^ | -0.5118^*^ |

AIC: Akaike information criterion; SC: Schwartz criterion. ^*^p<0.05; ^**^p<0.01.

TABLE S6 The association between health resources and services and under-5 mortality rate in West African countries, 2010-2019: estimated from spatial lag model in sensitive analysis.

| Independent variables | 2010 | | 2011 | | 2012 | | 2013 | | 2014 | |
| --- | --- | --- | --- | --- | --- | --- | --- | --- | --- | --- |
|  | coefficient | p-value | coefficient | p-value | coefficient | p-value | coefficient | p-value | coefficient | p-value |
| HDI | -0.1004 | <0.0001^**^ | -0.1112 | <0.0001^**^ | -0.0945 | <0.0001^**^ | -0.0729 | <0.0001^**^ | -0.0927 | <0.0001^**^ |
| Phy | -0.0025 | 0.3481 | -0.0018 | 0.0095^**^ | -0.0044 | <0.0001^**^ | -0.0015 | <0.0001^**^ | 0.0048 | <0.0001^**^ |
| Nam | -0.0019 | <0.0001^**^ | -0.0012 | <0.0001^**^ | -0.0010 | <0.0001^**^ | -0.0011 | <0.0001^**^ | -0.0016 | <0.0001^**^ |
| Php | 0.0036 | <0.0001^**^ | 0.0019 | <0.0001^**^ | 0.0006 | 0.0149^*^ | 0.0001 | 0.2838 | 0.0054 | <0.0001^**^ |
| Hc | 0.0001 | 0.0374^*^ | 0.0002 | <0.0001^**^ | 0.0004 | <0.0001^**^ | 0.0003 | <0.0001^**^ | 0.0000 | 0.4850 |
| CHE | 0.0004 | 0.0000^**^ | 0.0003 | <0.0001^**^ | 0.0003 | <0.0001^**^ | 0.0002 | <0.0001^**^ | 0.0000 | 0.8026 |
| Dtp | -0.0003 | 0.0717 | 0.0002 | <0.0001^**^ | 0.0005 | <0.0001^**^ | 0.0006 | <0.0001^**^ | 0.0004 | <0.0001^**^ |
| Mea | -0.0003 | <0.0001^**^ | -0.0002 | <0.0001^**^ | -0.0003 | <0.0001^**^ | -0.0006 | <0.0001^**^ | -0.0006 | <0.0001^**^ |
| Br | -0.0003 | 0.0046^**^ | -0.0001 | 0.0012^**^ | 0.0001 | <0.0001^**^ | -0.0001 | <0.0001^**^ | -0.0002 | <0.0001^**^ |
| Ant | -0.0012 | <0.0001^**^ | -0.0007 | <0.0001^**^ | -0.0003 | <0.0001^**^ | -0.0006 | <0.0001^**^ | -0.0006 | <0.0001^**^ |
| Con | -0.0001 | 0.0662 | -0.0001 | 0.0004^**^ | -0.0001 | <0.0001^**^ | -0.0001 | <0.0001^**^ | -0.0001 | 0.0048^**^ |
| Shp | 0.0004 | <0.0001^**^ | 0.0003 | <0.0001^**^ | 0.0002 | <0.0001^**^ | 0.0004 | <0.0001^**^ | 0.0004 | <0.0001^**^ |

^*^p<0.05; ^**^p<0.01.

Continued:

| Independent variables | 2015 | | 2016 | | 2017 | | 2018 | | 2019 | |
| --- | --- | --- | --- | --- | --- | --- | --- | --- | --- | --- |
|  | coefficient | p-value | coefficient | p-value | coefficient | p-value | coefficient | p-value | coefficient | p-value |
| HDI | -0.0876 | <0.0001^**^ | -0.1094 | <0.0001^**^ | -0.1139 | <0.0001^**^ | -0.0601 | 0.0432^*^ | -0.0813 | <0.0001^**^ |
| Phy | 0.0017 | 0.0001^**^ | 0.0015 | 0.0321^*^ | 0.0012 | 0.5076 | 0.0034 | 0.0344^*^ | 0.0025 | 0.0215^**^ |
| Nam | -0.0011 | <0.0001^**^ | -0.0002 | 0.5976 | -0.0005 | 0.3393 | -0.0004 | 0.1246 | -0.0002 | 0.3263 |
| Php | 0.0053 | <0.0001^**^ | 0.0027 | 0.1084 | 0.0058 | 0.1021 | 0.0054 | 0.0020^**^ | 0.0045 | 0.0001^**^ |
| Hc | 0.0001 | 0.0002^**^ | 0.0001 | 0.0444^**^ | 0.0000 | 0.7114 | 0.0002 | 0.0324^*^ | 0.0002 | 0.0088^**^ |
| CHE | 0.0000 | 0.0020^**^ | 0.0000 | 0.6357 | 0.0000 | 0.8447 | -0.0003 | 0.0752 | -0.0002 | 0.0647 |
| Dtp | 0.0007 | <0.0001^**^ | 0.0004 | 0.0005^**^ | 0.0007 | 0.2008 | 0.0002 | 0.3336 | 0.0005 | <0.0001^**^ |
| Mea | -0.0005 | <0.0001^**^ | -0.0001 | 0.5045 | -0.0005 | 0.4002 | -0.0002 | 0.5121 | -0.0002 | 0.0672 |
| Br | 0.0000 | 0.7600 | 0.0000 | 0.5486 | 0.0001 | 0.2685 | 0.0001 | 0.0443^*^ | 0.0002 | 0.0015^**^ |
| Ant | -0.0002 | 0.0074^**^ | -0.0002 | 0.2877 | -0.0002 | 0.3697 | -0.0001 | 0.4936 | 0.0000 | 0.9377 |
| Con | 0.0000 | 0.8154 | 0.0001 | 0.2520 | 0.0000 | 0.9137 | 0.0005 | 0.0318^*^ | 0.0004 | 0.0110^**^ |
| Shp | 0.0002 | <0.0001^**^ | 0.0002 | 0.0246^*^ | 0.0001 | 0.4499 | -0.0002 | 0.3117 | -0.0001 | 0.3579 |

^*^p<0.05; ^**^p<0.01.

TABLE S7 The association between health resources and services and under-5 mortality rate in West African countries, 2010-2019: estimated from spatial error model in sensitive analysis.

| Independent variables | 2010 | | 2011 | | 2012 | | 2013 | | 2014 | |
| --- | --- | --- | --- | --- | --- | --- | --- | --- | --- | --- |
|  | coefficient | p-value | coefficient | p-value | coefficient | p-value | coefficient | p-value | coefficient | p-value |
| HDI | -0.0844 | <0.0001^**^ | -0.1152 | <0.0001^**^ | -0.1048 | <0.0001^**^ | -0.1508 | <0.0001^**^ | -0.1706 | <0.0001^**^ |
| Phy | -0.0008 | 0.2266 | -0.0017 | <0.0001^**^ | -0.0040 | <0.0001^**^ | 0.0097 | <0.0001^**^ | 0.0041 | 0.0045^**^ |
| Nam | -0.0019 | <0.0001^**^ | -0.0012 | <0.0001^**^ | -0.0008 | <0.0001^**^ | -0.0035 | <0.0001^**^ | -0.0008 | 0.2424 |
| Php | 0.0052 | <0.0001^**^ | 0.0023 | <0.0001^**^ | 0.0007 | 0.0150^*^ | 0.0125 | <0.0001^**^ | 0.0053 | 0.0112^*^ |
| Hc | 0.0001 | <0.0001^**^ | 0.0002 | <0.0001^**^ | 0.0002 | 0.0023^**^ | 0.0003 | <0.0001^**^ | 0.0000 | 0.8532 |
| CHE | 0.0002 | <0.0001^**^ | 0.0002 | <0.0001^**^ | 0.0003 | <0.0001^**^ | -0.0002 | <0.0001^**^ | 0.0001 | 0.1743 |
| Dtp | 0.0000 | 0.8794 | 0.0002 | 0.0004^**^ | 0.0005 | <0.0001^**^ | 0.0014 | <0.0001^**^ | -0.0002 | 0.2520 |
| Mea | -0.0004 | <0.0001^**^ | -0.0002 | <0.0001^**^ | -0.0004 | <0.0001^**^ | -0.0029 | <0.0001^**^ | -0.0003 | 0.1882 |
| Br | -0.0003 | <0.0001^**^ | -0.0001 | 0.0017^**^ | 0.0000 | 0.3379 | -0.0015 | <0.0001^**^ | -0.0005 | 0.0004^**^ |
| Ant | -0.0011 | <0.0001^**^ | -0.0007 | <0.0001^**^ | -0.0005 | <0.0001^**^ | -0.0039 | <0.0001^**^ | -0.0015 | <0.0001^**^ |
| Con | -0.0002 | 0.0001^**^ | -0.0001 | 0.0926 | -0.0002 | <0.0001^**^ | -0.0004 | <0.0001^**^ | -0.0004 | 0.0076 ^**^ |
| Shp | 0.0004 | <0.0001^**^ | 0.0003 | <0.0001^**^ | 0.0002 | <0.0001^**^ | 0.0023 | <0.0001^**^ | 0.0008 | <0.0001^**^ |

^*^p<0.05; ^**^p<0.01.

Continued:

| Independent variables | | 2015 | | 2016 | | 2017 | | 2018 | | 2019 | |
| --- | --- | --- | --- | --- | --- | --- | --- | --- | --- | --- | --- |
|  |  | coefficient | p-value | coefficient | p-value | coefficient | p-value | coefficient | p-value | coefficient | p-value |
| HDI | -0.0846 | | <0.0001^**^ | -0.0864 | <0.0001^**^ | -0.1176 | <0.0001^**^ | -0.1205 | <0.0001^**^ | 0.0573 | 0.0240^*^ |
| Phy | 0.0031 | | <0.0001^**^ | 0.0000 | 0.9790 | 0.0015 | 0.3471 | 0.0016 | 0.2037 | -0.0009 | 0.3638 |
| Nam | -0.0024 | | <0.0001^**^ | -0.0009 | 0.1228 | 0.0013 | 0.2334 | -0.0004 | 0.0093^**^ | -0.0017 | <0.0001^**^ |
| Php | 0.0116 | | <0.0001^**^ | 0.0057 | 0.0288^*^ | -0.0047 | 0.3830 | 0.0074 | <0.0001^**^ | 0.0094 | <0.0001^**^ |
| Hc | 0.0000 | | 0.1117 | 0.0000 | 0.3907 | 0.0002 | 0.1197 | 0.0002 | <0.0001^**^ | -0.0001 | 0.3770 |
| CHE | 0.0000 | | 0.5247 | 0.0000 | 0.4949 | -0.0004 | 0.0111^*^ | -0.0001 | 0.3141 | -0.0004 | 0.0001^**^ |
| Dtp | 0.0011 | | <0.0001^**^ | 0.0002 | 0.2537 | -0.0007 | 0.3185 | 0.0006 | 0.0001^**^ | 0.0010 | <0.0001^**^ |
| Mea | -0.0012 | | <0.0001^**^ | -0.0003 | 0.4007 | 0.0012 | 0.1149 | -0.0003 | 0.0169^*^ | -0.0006 | <0.0001^**^ |
| Br | -0.0003 | | <0.0001^**^ | 0.0000 | 0.6261 | 0.0000 | 0.9325 | 0.0000 | 0.8077 | 0.0005 | <0.0001^**^ |
| Ant | -0.0008 | | <0.0001^**^ | -0.0008 | 0.0060^**^ | -0.0002 | 0.2794 | 0.0000 | 0.6636 | 0.0008 | 0.0001^**^ |
| Con | -0.0002 | | 0.0561 | 0.0002 | 0.0200^*^ | 0.0013 | <0.0001^**^ | 0.0001 | 0.4383 | 0.0014 | <0.0001^**^ |
| Shp | 0.0006 | | <0.0001^**^ | 0.0001 | 0.3001 | -0.0002 | 0.1367 | 0.0001 | 0.5656 | -0.0010 | <0.0001^**^ |

*p<0.05; **p<0.01.

TABLE S8 The association between health resources and services and under-5 mortality rate in West African countries, 2010-2019: estimated from ordinary least square in sensitive analysis.

| Independent variables | 2010 | | 2011 | | 2012 | | 2013 | | 2014 | |
| --- | --- | --- | --- | --- | --- | --- | --- | --- | --- | --- |
|  | coefficient | p-value | coefficient | p-value | coefficient | p-value | coefficient | p-value | coefficient | p-value |
| HDI | -0.0891 | 0.1468 | -0.1053 | 0.0057 | -0.0941 | 0.0117 | -0.0806 | 0.1431 | -0.0880 | 0.1485 |
| Phy | -0.0053 | 0.3877 | -0.0028 | 0.0560 | -0.0048 | 0.0329 | -0.0031 | 0.2848 | 0.0014 | 0.6055 |
| Nam | -0.0017 | 0.1113 | -0.0012 | 0.0079 | -0.0010 | 0.0207 | -0.0011 | 0.2095 | -0.0018 | 0.2668 |
| Php | 0.0034 | 0.2697 | 0.0016 | 0.1314 | 0.0007 | 0.4165 | 0.0011 | 0.6202 | 0.0066 | 0.1951 |
| Hc | 0.0002 | 0.2578 | 0.0002 | 0.0155 | 0.0004 | 0.0161 | 0.0002 | 0.2063 | 0.0000 | 0.8528 |
| CHE | 0.0004 | 0.2599 | 0.0003 | 0.0123 | 0.0003 | 0.0149 | 0.0002 | 0.1117 | 0.0000 | 0.5255 |
| Dtp | -0.0001 | 0.8676 | 0.0003 | 0.0644 | 0.0005 | 0.0226 | 0.0004 | 0.2329 | 0.0002 | 0.5218 |
| Mea | -0.0002 | 0.3092 | -0.0002 | 0.0357 | -0.0003 | 0.0861 | -0.0003 | 0.3823 | -0.0004 | 0.3502 |
| Br | -0.0001 | 0.4079 | 0.0000 | 0.3373 | 0.0001 | 0.1583 | 0.0000 | 0.7960 | -0.0002 | 0.5365 |
| Ant | -0.0010 | 0.1287 | -0.0007 | 0.0098 | -0.0003 | 0.1120 | -0.0005 | 0.2732 | -0.0007 | 0.2609 |
| Con | -0.0001 | 0.6968 | 0.0000 | 0.3484 | -0.0001 | 0.2076 | -0.0001 | 0.7495 | 0.0001 | 0.7836 |
| Shp | 0.0003 | 0.2298 | 0.0003 | 0.0207 | 0.0002 | 0.0871 | 0.0002 | 0.2647 | 0.0003 | 0.4037 |

Continued:

| Independent variables | 2015 | | 2016 | | 2017 | | 2018 | | 2019 | |
| --- | --- | --- | --- | --- | --- | --- | --- | --- | --- | --- |
|  | coefficient | p-value | coefficient | p-value | coefficient | p-value | coefficient | p-value | coefficient | p-value |
| HDI | -0.0818 | 0.1000 | -0.0937 | 0.1733 | -0.0973 | 0.3636 | -0.0629 | 0.4708 | -0.0689 | 0.2929 |
| Phy | 0.0006 | 0.6616 | 0.0003 | 0.9050 | 0.0010 | 0.8703 | 0.0035 | 0.5104 | 0.0023 | 0.5429 |
| Nam | -0.0015 | 0.1480 | -0.0009 | 0.4176 | -0.0008 | 0.6808 | -0.0003 | 0.6401 | -0.0004 | 0.4460 |
| Php | 0.0068 | 0.1019 | 0.0061 | 0.2493 | 0.0075 | 0.5478 | 0.0050 | 0.3369 | 0.0060 | 0.1193 |
| Hc | 0.0001 | 0.4990 | 0.0001 | 0.7560 | 0.0000 | 0.9093 | 0.0002 | 0.5091 | 0.0002 | 0.4785 |
| CHE | 0.0000 | 0.3866 | 0.0000 | 0.8350 | -0.0001 | 0.9480 | -0.0003 | 0.5546 | -0.0003 | 0.4902 |
| Dtp | 0.0006 | 0.1332 | 0.0004 | 0.3317 | 0.0005 | 0.7750 | 0.0002 | 0.7659 | 0.0005 | 0.2245 |
| Mea | -0.0005 | 0.1952 | -0.0003 | 0.5461 | -0.0004 | 0.8301 | -0.0001 | 0.8535 | -0.0003 | 0.4856 |
| Br | 0.0000 | 0.9259 | 0.0000 | 0.9590 | 0.0001 | 0.7754 | 0.0001 | 0.5103 | 0.0002 | 0.3266 |
| Ant | -0.0003 | 0.3896 | -0.0004 | 0.4327 | -0.0003 | 0.6822 | -0.0001 | 0.8386 | 0.0000 | 0.9660 |
| Con | 0.0001 | 0.6924 | 0.0001 | 0.6582 | 0.0002 | 0.8679 | 0.0005 | 0.4968 | 0.0005 | 0.3781 |
| Shp | 0.0002 | 0.3622 | 0.0001 | 0.6995 | 0.0000 | 0.9668 | -0.0001 | 0.7151 | -0.0002 | 0.5583 |
